# PRGF as adjunct to DBB in maxillary sinus floor augmentation: histological results of a pilot split-mouth study

**DOI:** 10.1186/s40729-019-0166-6

**Published:** 2019-04-01

**Authors:** Leonidas Batas, Lazaros Tsalikis, Andreas Stavropoulos

**Affiliations:** 1 Department of Preventive Dentistry, Periodontology and Implant Biology, School of Dentistry, Aristotle University of Thessaloniki, Thessaloniki, Greece; 2 Department of Periodontology, Faculty of Odontology, Malmö University, Malmö, Sweden; 3 Center for Experimental and Preclinical Biomedical Research (CEPBR), Athens, Greece; 4 Division of Conservative Dentistry and Periodontology, University Clinic of Dentistry, Medical University of Vienna, Vienna, Austria

**Keywords:** PRGF, Deproteinized bovine bone, Sinus elevation, Histology, Humans

## Abstract

**Background:**

Various technologies of autologous blood concentrates are currently evaluated for their potential to enhance bone formation.

**Aim:**

To report on the histological outcome of maxillary sinus floor augmentation (MSFA) with deproteinized bovine bone (DBB) in combination with chair-side prepared autologous platelet-rich growth factor (PRGF), in comparison to that with DBB alone.

**Materials and methods:**

Six partially edentulous patients with ≤ 3-mm residual bone height bilaterally in the posterior maxilla were subjected to MSFA with the lateral window technique, using DBB in combination with PRGF (PRGF System1 Vitoria, Spain) on one side or DBB alone on the contralateral side. Cylindrical biopsies from the augmented sinuses were collected during implant installation, ca. 6 months post-MSFA, and subjected to non-decalcified histological and histomorphometric evaluation.

**Results:**

The collected biopsies varied in length (range 3.5–9.9 mm); consequently, the portion of the biopsy representing augmented tissues also varied (range 2.3–14.6 mm^2^). New bone formation with a trabecular appearance and numerous DBB particles in contact with the new bone or with loose connective tissue were observed. No differences in the relative volumes of bone formation were found in sinuses augmented with DBB + PRGF or DBB alone 6 months after MSFA (35.6 ± 8.26 mm and 37.8 ± 3.15 mm, respectively).

**Conclusion and clinical implications:**

In conclusion, based on these preliminary results, PRGF as adjunct to DBB for MSFA, except from improved handling during the operation, does not appear to enhance nor interfere with bone formation inside the human sinus 6 months after MSFA, compared with the use of DBB alone.

## Introduction

Maxillary sinus floor augmentation (MSFA) is a standard procedure to re-establish adequate bone volume and ridge height for implant installation in the posterior maxilla. MSFA was introduced to the profession by Boyne in 1980 [1–3] and is a highly predictable technique with high graft and implant survival rates [4, 5]. Initially, sinus elevation techniques relied on grafting with 100% autogenous bone, harvested from either oral (ramus, chin) or extraoral (iliac crest) donor sites [6–8]. It was subsequently found that autogenous bone grafting can be replaced, in whole or in part, by a variety of bone substitutes, like allografts, xenografts, alloplasts, [9–11], alone, or in combination with growth factors [12–16].

During recent years, several technologies of chair-side, autologous blood concentrates have been proposed as growth factor (GF) sources [17]. These autologous blood concentrates consist of a volume of plasma enriched with a large number of platelets (i.e., platelet-rich constructs) which, after activation, they release GFs (e.g., platelet-derived growth factor (PDGF), transforming growth factor beta (TGF-b), insulin-like growth factor (IGF), vascular endothelial growth factor (VEGF), basic fibroblast growth factor (bFGF), and hepatocyte growth factors (HGF), with the potential to enhance bone healing/regeneration [13, 17–19, 20]. Plasma-rich in growth factors (PRGF) [21] is such autologous blood-derived concentrate and has been described to enhance bone formation in bone defects, including MSFA [21–24].

Nevertheless, PRGF has a gel-like consistency and thus lacks space-provision capacities; hence, it seems reasonable that in MSFA, PRGF needs to be combined with a space providing material, e.g., a bone substitute, in order to achieve adequate volume of augmentation. Among the most commonly used bone substitutes in oral surgical procedures is deproteinized bovine bone (DBB) [25, 26], and successful (histological and clinical) outcomes have been reported for MSFA with DBB alone or in combination with autogenous bone, cells, and/or growth factors [27–29]. In one human histological case series with five patients [30], PRGF + DBB resulted in increased vascularization and increased bone formation 5 months after MSFA compared with only DBB implantation.

Thus, the aim of the present report was to add information on the existing, rather scarce, evidence about the histological outcome of MSFA with DBB in combination with chair-side prepared PRGF compared with that of MSFA with DBB alone.

## Material methods

Six patients, requiring bilateral sinus augmentation procedures prior to placement of implants and no contraindications for this procedure (e.g., uncontrolled diabetes, long-term steroid usage, and blood disorders) were selected from those presenting at the Aristotle University of Thessaloniki School of Dentistry, Department of Periodontology. All patients were advised of alternative treatment plans and selected the plan requiring maxillary sinus elevation. Further, the patients were informed of the requirements for participation in the study, and all had the option of withdrawing from the study at any time. The nature of the study was explained to each patient, and each signed an informed consent form that was approved by the University ethical Committee on Activities Involving Human Subjects of the University of Thessaloniki, Dental School (14/02-02-2017). Due to the pilot character of this study, no sample size or power calculation was performed and only a few patients in need for a bilateral sinus augmentation were included. The intention was to assess any trends for differences between groups, and thus to decide on the possible relevance of a larger-scale study.

### Surgical procedure

**Fig. 1:**
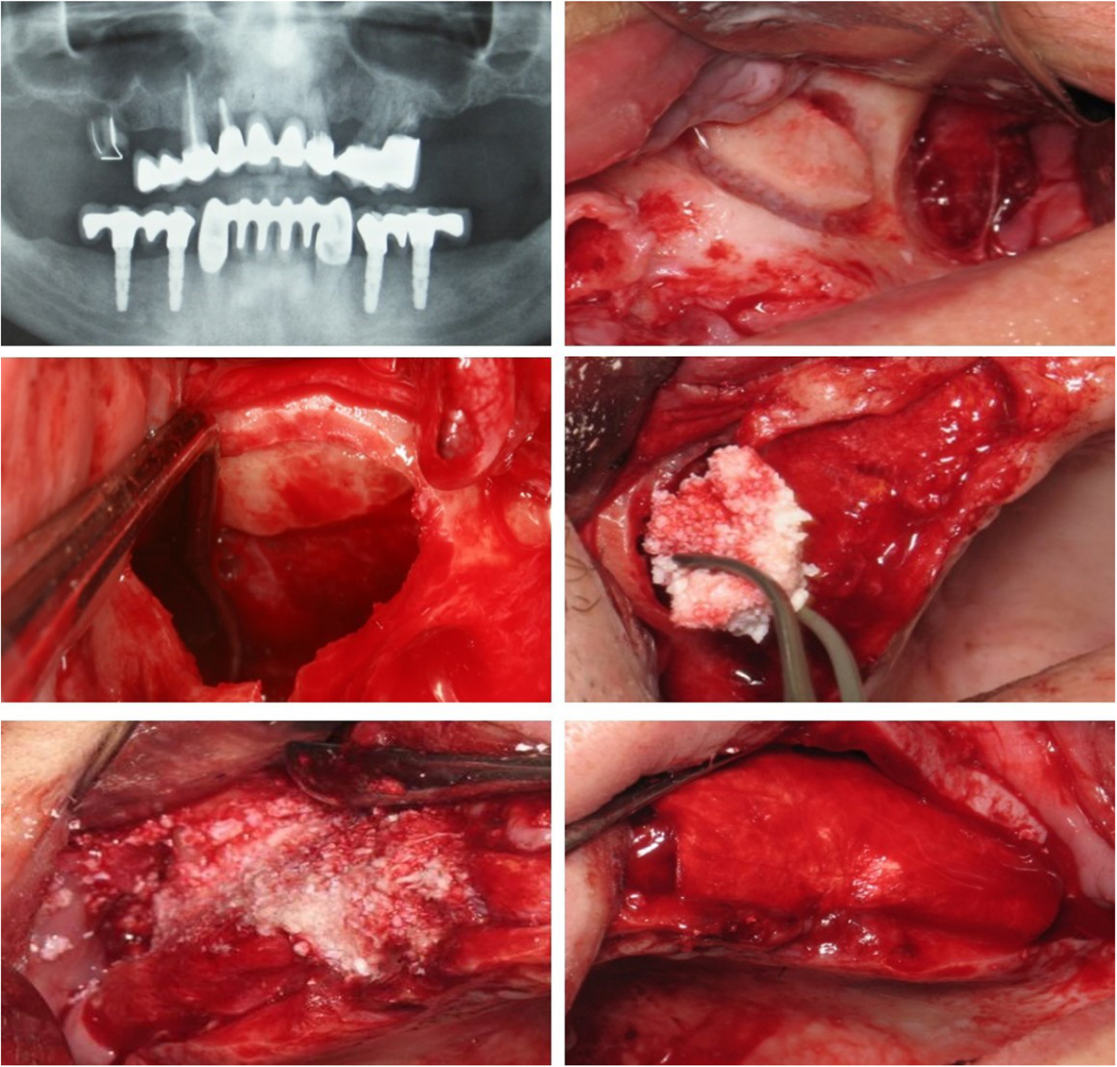
Panoramic radiograph (**a**) of one patient receiving bi-lateral MSFA with the lateral window approach (**b**), where after sinus membrane elevation (**c**), one site was grafted with DBB + PRGF (**d**) and the other with DBB alone (**e**); the window was always covered with a collagen membrane

On the day of surgery, a flip of a coin determined the side receiving PRGF (PRGF System 1, Vitoria, Spain) in combination with DBB (Bio-Oss/Geistlich Biomaterials, Switzerland); the contralateral side received only DBB. Briefly, MSFA was performed with a lateral window approach, where after osteotomy, the lateral bone window was lifted upwards together with the Schneiderian membrane very carefully separated from the bone, and the bone graft material was placed into the newly created space (Fig. 1). All 12 sinuses in this pilot study were grafted with cancellous DBB particles 0.25 to 1 mm in size.

### PRGF preparation

**Fig. 2:**
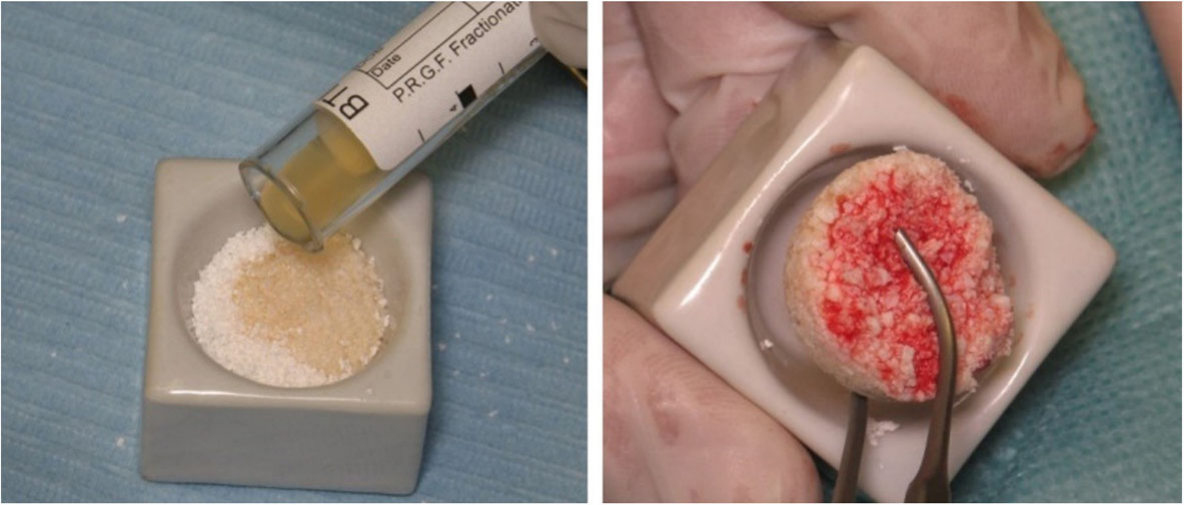
PRGF is mixed with DBB (**a**) and after a few minutes the mixture attains a viscous consistency facilitating easy handling of the bone substitute particles (**b**)

Twenty milliliters of peripheral blood was collected by venipuncture directly into tubes (BTI blood collecting tubes, BTI Vitoria, Spain) containing 3.8% (wt/vol) sodium citrate as anticoagulant. The blood was then centrifuged at 1400 rpm for 8 min at room temperature (PRGF System1, Vitoria, Spain), and thus separated into its three basic components: red blood cells (at the bottom of the tube); PRGF (in the middle of the tube); and plasma poor in growth factors (at the upper part of the tube). The 0.5 ml PRGF fraction located just above the red cell fraction was collected, with care being taken to avoid the overlying buffy coat, and deposited in an Eppendorf tube. Clotting and activation were initiated by adding 50 μl calcium chloride solution (10% *w*/*v*) to the Eppendorf tube. The activated PRGF was then mixed with 1 cc of DBB in a glass dish, and after 5–8 min the material became viscous and was ready for use (Fig. 2).

### Infection and pain control

All patients were prescribed systemic antibiotics (2 g amoxicillin with clavulanic acid) starting 1 day before surgery and for 6 days post-surgically. Dexamethasone (8 mg) was prescribed and administered orally right before the surgery and for the following 3 days with a decreasing dose (8, 4, and 2 mg respectively). Analgesics were also prescribed (600mgr ibuprofen) for pain control. The patients were then asked to describe the post-operative discomfort as no pain discomfort, pain controlled with painkillers, or great pain.

### Histologic and histomorphometric analyses

**Fig. 3:**
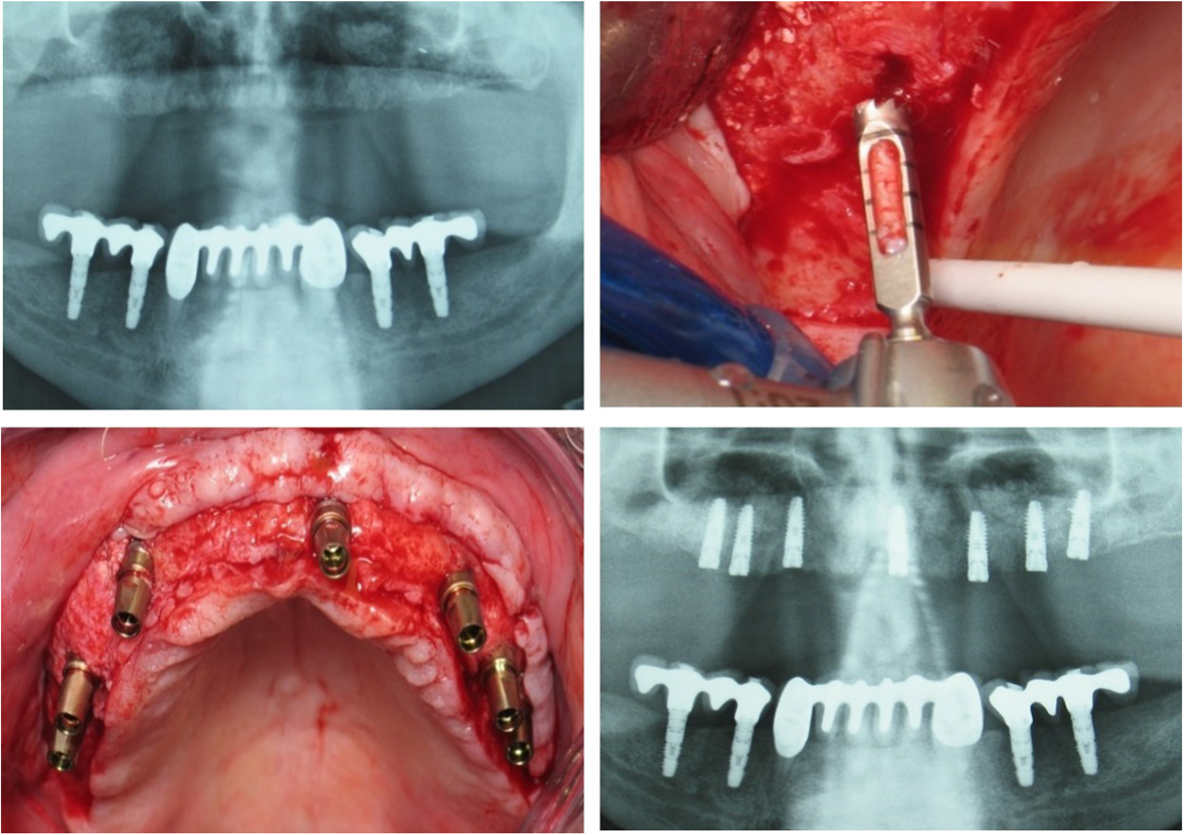
Panoramic X-ray of the case presented in Fig. 2, 6 months post-operatively (**a**), showing increased radio-opacity in both grafted sinuses. Biopsies were obtained with trephine burs during oral implant site preparation (**b**)

**Fig. 4:**
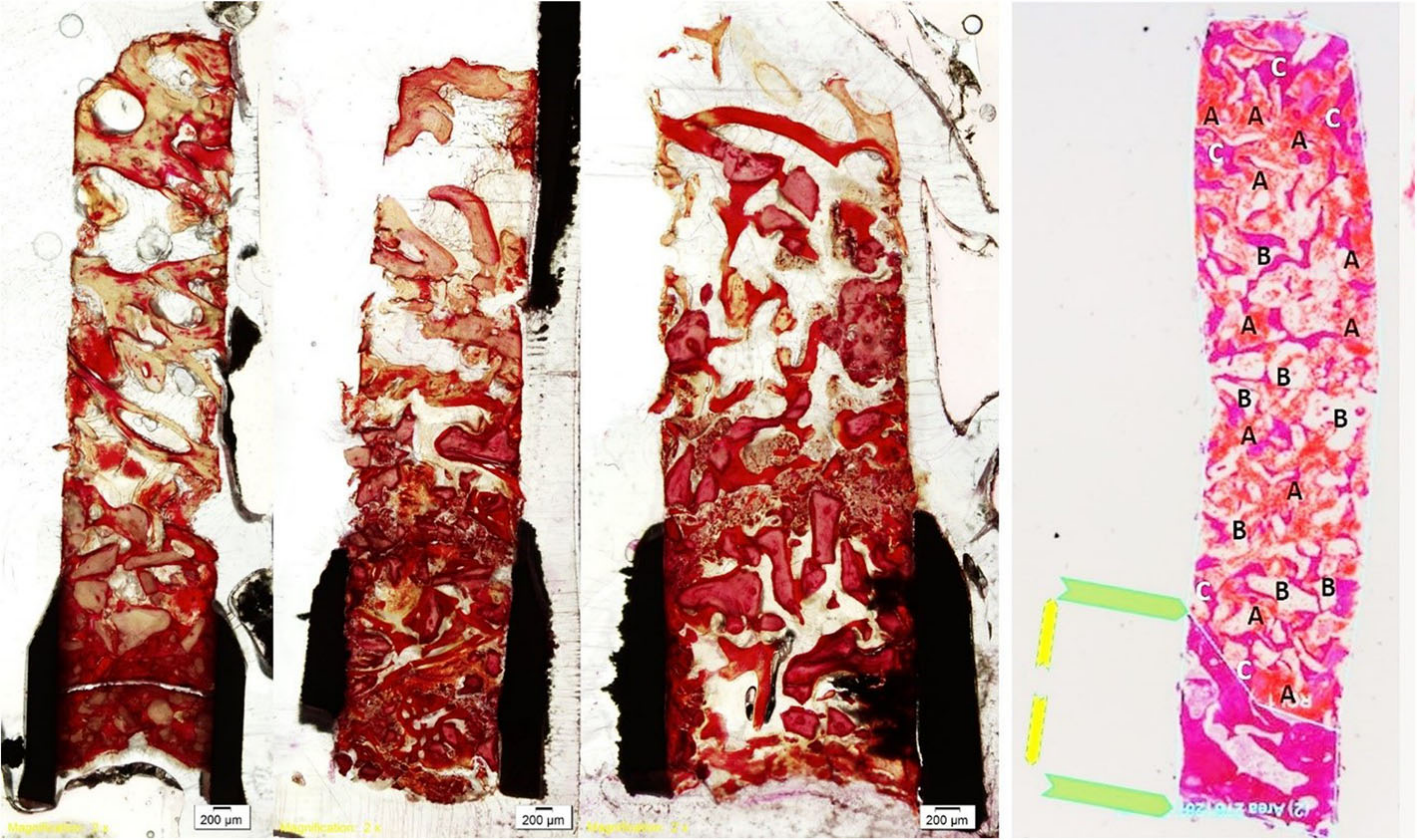
The margin of pristine bone and augmented tissues was identified, and the various tissue components within the augmented area were semi-automatically estimated by dedicated image analysis software. Newly formed bone, connective tissue, DBB, within the augmented area, were identified and calculated

Cylindrical biopsies were retrieved at the time of implant placement by means of trephine burs with an external/internal diameter of 3 mm/2 mm, about 6 months after MSFA (Fig. 3). Immediately after harvesting, the trephines containing the tissue cores were placed in 70% alcohol for fixation. The cores were code-masked to facilitate blind histological assessment. After 2 weeks, the biopsy core was removed from the trephine, whenever possible; otherwise, the entire trephine-biopsy complex was further processed for non-decalcified sectioning, including dehydration in a series of increasing concentrations of ethanol and embedding in methylmethacrylate. Longitudinal sections were generated with a cutting-grinding technique and then stained with van Gieson’s picro-fuchsin. Histological and histomorphometrical analysis was performed while viewing the most central section of each biopsy under a microscope with incandescent light (BH-50, Olympus, Ballerup, Denmark) fitted to a video camera (Olympus DP70, Olympus). First, the margin between pristine bone and newly formed tissue inside the sinus was histologically identified, and then the area fractions (%) of newly formed bone (mineralized tissue and bone marrow), soft connective tissue, residual biomaterial, empty spaces, and debris were estimated by a semi-automated technique based on color segmentation through the software (Adobe Photoshop; Adobe) (Fig. 4).

Summary statistics were used to describe the data, and the two-sided Wilcoxon test for paired observations was used to evaluate differences in the various tissue components between the two groups. The significance level was set at *P* < 0.05. The SPSS 13.0 software (SPPS Inc., Chicago, IL, USA) was used for the statistical analysis.

## Results

Handling and placement of DBB into the sinus were considerably improved when combined with PRGF (Fig. 2). Further, most patients referred to increased pain and swelling associated with sites grafted with only DBB. No major adverse reactions were noted during MSFA or the post-operative phase, and no patients were excluded from the study. Further, all retrieved specimens could be processed and analyzed.

Variation in the length of the retrieved biopsy (range 3.5–9.9 mm) was observed, and thus the portion of the biopsy representing augmented tissues also varied among biopsies (range 2.3–14.6 mm^2^); however, this was not obviously related to the original height of the residual ridge, neither with the amount of new bone formation within the biopsy. The biopsies consisted of a part including the cortical and trabecular bone of the residual alveolar ridge and a part corresponding to the new tissues formed in the sinus cavity. The new tissues consisted of variable amount of trabecular bone, DBB particles, loose connective tissue, and occasionally bone marrow. The newly formed bone was often in contact with the DBB particles, while no obvious signs of inflammation or foreign body reaction were observed.

There was no statistically significant difference between the two groups regarding any of the various tissue components within the sinus. The relative volume of new bone formation in sinuses augmented with DBB + PRGF was 35.6 ± 8.26 mm and in sinuses augmented with DBB alone was 37.8 ± 3.15 mm (*P* > 0.05) (Table 1).

**Table 1:** Relative volume of new bone formation

	New bone (%)		Connective tissue (%)		Particulates DBB (%)	
*N* (patients)	PRGF + DBB	DBB	PRGF + DBB	DBB	PRGF + DBB	DBB
1	43.86	40.95	24.02	26.52	32.12	32.53
2	38.3	39.77	40.85	38.11	20.85	22.12
3	35.47	37.9	34.26	36.80	30.26	23.95
4	34.74	37.64	37.80	41.45	27.46	20.91
5	33.87	35.89	33.55	28.95	32.58	35.16
6	27.36	34.65	37.69	37.50	34.95	27.85
Mean	35.6	37.8	34.69	34.88	29.7	27.08
SD	8.26	3.15	5.86	5.81	5	5.79

Histomorphometric characterization of the percentages of newly formed bone, connective tissue, and DBB particulates for each patient

## Discussion

The results of this pilot study showed that PRGF as an adjunct to DBB grafting for MSFA did not enhance bone formation compared with DBB grafting alone, 6 months post-operatively. This result is directly in contrast with what presented in a report on a clinical study similar to the present one [30]. In this study, including five patients with MSFA, the same combination of PRGF + DBB resulted in increased vascularization and increased bone formation compared with only DBB implantation, 5 months post-op.

Indeed, conflicting results have been reported in both pre-clinical in vivo and clinical studies regarding the potential of PRGF to enhance bone regeneration. In several preclinical in vitro studies, bone defects treated with PRGF showed enhanced bone regeneration compared with controls. For example, PRGF implanted in extraction sockets, in humans, resulted in larger amounts of bone fill, compared with sockets left to heal alone [31], while narrow cylindrical defects in goat tibiae showed significantly larger amounts of mature bone trabeculae when treated with PRGF, than spontaneously healing sites [23]. In contrast, in other preclinical in vivo studies, adjunct use of PRGF did not promote bone regeneration or implant osseointegration, irrespective of the use or not of bone substitute materials, comparing to relevant controls [32–34]. Further, when PRGF was implanted in human extraction sockets, similar amounts of bone have been observed with those in spontaneously healed sockets [35, 36]. Similarly conflicting results have been reported regarding the potential of other autologous blood preparations to enhance bone regeneration (e.g., PRP) [18, 37].

PRGF is suggested to be superior to other technologies of autologous blood concentrates, because of the unique preparation method, which results in (a) a high platelet concentration within the separated plasma (three times more than in peripheral blood) without white cell contamination, (b) slow release of GF over 7 days, when in other autologous blood concentrates systems (e.g., PRP) the release stops within 1 h when thrombin is used [17, 38, 39], (c) a leukocyte-free homogenous fibrin matrix, with reduced levels of proinflammatory cytokines interleukin IL-1β and IL-16. The present pilot experiment, however, was not designed to evaluate superiority of PRGF over other types of autologous blood preparations, and lack of any significant differences between the PRGF and control group in this pilot experiment do not necessarily imply lack of effect of the PRGF technology in MSFA procedures. Lack of additional effect of other types of growth factors in terms of bone regeneration when used as adjuncts to bone substitutes in MSFA has been observed in similar histological studies with similar or longer observation times as herein [11, 14, 40, 41]. For instance, in a study on bilateral MSFA, comparing DBB + PRP vs DBB alone, no benefit of the combined approach was observed [40], and in a recent systematic review of RCTs on sinus lift with or without adjunct use of PRP [42], the majority of studies failed to show a significant additive effect of PRP. In this context, the time point of post-op evaluation is critical in terms of interpreting the results of studies on biomaterials/adjuncts to enhance bone formation, i.e., extended observation times, may “wash-out” any possible positive effect of adjuncts on healing. Indeed, it appears that the major bulk of regenerated bone in sinuses grafted with bone substitutes forms within the first 4–5 months, and thereafter only relatively small increase in bone formation may be observed [43, 44]. In the same line, in a recently published systematic review of histomorphometric data from human biopsies, sinuses grafted with autogenous bone and PRP showed larger average amounts of bone formation compared to sinuses grafted with autogenous bone alone, only at early observation times (i.e., ≤ 4.5 months) and not at later time-points [45]. The differences between the abovementioned study [24] and the present one may thus be rather related to anatomical variation in sinus dimensions (e.g., width) [46] and/to the method of histomorphometric analysis, rather than in the difference in the time-frame of healing (1 month).

In conclusion, based on these preliminary results, PRGF as adjunct to DBB for MSFA, except from improved handling during the operation, does not appear to enhance nor interfere with bone formation inside the human sinus 6 months after MSFA, compared with the use of DBB alone.
